# Timing of Creatine Supplementation around Exercise: A Real Concern?

**DOI:** 10.3390/nu13082844

**Published:** 2021-08-19

**Authors:** Felipe Ribeiro, Igor Longobardi, Pedro Perim, Breno Duarte, Pedro Ferreira, Bruno Gualano, Hamilton Roschel, Bryan Saunders

**Affiliations:** 1 Applied Physiology and Nutrition Research Group, School of Physical Education and Sport, Rheumatology Division, Faculdade de Medicina FMUSP, University of São Paulo, São Paulo 01246-903, SP, Brazil; felipe.is2@hotmail.com (F.R.); i.long@usp.br (I.L.); pedroperim13@gmail.com (P.P.); brenoduarte@usp.br (B.D.); pedro.h.a.ferreira95@gmail.com (P.F.); gualano@usp.br (B.G.); hars@usp.br (H.R.); 2 Centro Universitário São Camilo, São Paulo 04263-200, SP, Brazil; 3 Food Research Center (FoRC), University of São Paulo, São Paulo 05508-080, SP, Brazil; 4 Institute of Orthopaedics and Traumatology, Faculty of Medicine FMUSP, University of São Paulo, São Paulo 01246-903, SP, Brazil

**Keywords:** dietary supplements, ergogenic aid, hypertrophy, resistance training, sports nutrition, strength, supplementation

## Abstract

Creatine has been considered an effective ergogenic aid for several decades; it can help athletes engaged in a variety of sports and obtain performance gains. Creatine supplementation increases muscle creatine stores; several factors have been identified that may modify the intramuscular increase and subsequent performance benefits, including baseline muscle Cr content, type II muscle fibre content and size, habitual dietary intake of Cr, aging, and exercise. Timing of creatine supplementation in relation to exercise has recently been proposed as an important consideration to optimise muscle loading and performance gains, although current consensus is lacking regarding the ideal ingestion time. Research has shifted towards comparing creatine supplementation strategies pre-, during-, or post-exercise. Emerging evidence suggests greater benefits when creatine is consumed after exercise compared to pre-exercise, although methodological limitations currently preclude solid conclusions. Furthermore, physiological and mechanistic data are lacking, in regard to claims that the timing of creatine supplementation around exercise moderates gains in muscle creatine and exercise performance. This review discusses novel scientific evidence on the timing of creatine intake, the possible mechanisms that may be involved, and whether the timing of creatine supplementation around exercise is truly a real concern.

## 1. Introduction

Athletes (and physically active individuals) are interested in nutritional strategies that are aimed at enhancing exercise performance. Creatine (Cr) deserves a special place among the plethora of ergogenic supplements, as it is one of the most studied and scientifically supported supplements on the market [1,2]. Creatine is a naturally occurring non-protein nitrogen compound synthesised in the liver and kidney from precursor amino acids, arginine, glycine, and methionine. Most of the body’s Cr is found in muscle (95%), of which two-thirds are stored as phosphorylcreatine (PCr), the remaining third as free Cr [3], with less than 5% found in other tissues, such as the brain and testes [4]. In a seminal study by Harris et al. (1992), it was demonstrated for the first time in humans that Cr supplementation, at varying doses of 20–30 g/day, ingested over several individual 5 g doses throughout the day, could increase total intramuscular Cr content (TCr = PCr + Cr) by as much as 20% [5]. Numerous subsequent studies have shown the efficacy of Cr supplementation in increasing muscle Cr content, including using more gradual loading protocols [6,7,8].

Several factors could influence the individual intramuscular increase in TCr and subsequent performance benefits as a consequence of Cr supplementation, including baseline muscle Cr content, type II muscle fibre content and size, and habitual dietary intake of Cr and aging [9,10]. Interestingly, it has been known for some time that exercise can enhance Cr loading in muscle [5], and the specific timing of Cr supplementation in relation to exercise has more recently been touted as an important consideration, in order to optimise training gains, although the current consensus on its importance is lacking. Emerging evidence suggests that post-exercise Cr ingestion may provide superior benefits compared to pre-exercise consumption [11,12], although several methodological limitations presented by these investigations currently preclude definitive interpretations of these results.

The purpose of this narrative review is to summarise and discuss current evidence and new emerging questions on the influence of Cr supplementation timing, in regard to exercise, on muscle Cr content and physical performance.

## 2. Creatine Supplementation

Studies show that creatine supplementation in doses of 5–20 g/day for >5 days can increase intramuscular Cr and PCr to the point of saturation [8,13]. This increase in PCr is associated with the main mechanism of action, regarding the ergogenic effect of Cr supplementation [14]. Phosphorylcreatine can provide an inorganic phosphate (Pi) molecule for the resynthesis of ATP via the Cr kinase reaction, in which Pi donation from PCr degradation is used by adenosine diphosphate (ADP) and, consequently, increases ATP resynthesis. Creatine phosphokinase is the enzyme that catalyses this reaction and is limited only by the concentration of its substrates and products, namely Cr and PCr [15]. This phosphagen system, also termed the ATP-CP system, is the fastest way to supply ATP for skeletal muscle metabolism [16]. Precisely for this reason, the ATP-CP system is related to high-intensity and short-duration exercises [17] and, thus, is associated with greater total work capacity [1]. In this respect, the ATP-CP system serves as an important regulator of muscle metabolism, which explains the ergogenic benefits of Cr supplementation throughout training. Enhancing the capacity of ATP resynthesis should increase available energy during exercise, prolonging the work capacity of the skeletal muscles, delaying the onset of muscle fatigue, and improving performance.

Strong scientific evidence suggests that Cr can lead to beneficial improvements in exercise performance; however, there also appears to be some variations in the response to Cr supplementation due to a number of factors, which will be presented in the next section.

### Factors Modifying the Effect of Creatine Supplementation on Muscle Creatine Content

Several factors have been shown to modify the effects of Cr supplementation on muscle Cr content. Daily dose and duration play important roles in how quickly and how much Cr stores are increased. Five to seven days of supplementation with a daily dose of 20 g·day^−1^ is sufficient to saturate muscle creatine stores [5], which is approximately 140 to 160 mmol·kg^−1^ of dry muscle. This has become a commonly employed dose in the literature and termed the “loading phase”. Nonetheless, a more gradual dosing strategy of 3 g·day^−1^ leads to similar increases, but over a longer period (~28 days; [8]). Greater increases in muscle Cr are shown in those with lower initial muscle Cr content [5], while carbohydrate co-supplementation may increase Cr uptake via insulin-mediated stimulation [6,18] of the Cr transporter, CreaT. Although this mechanism of insulin stimulated Cr uptake remains to be mechanistically confirmed, if it holds true, this will only be relevant within the first few days at high doses (e.g., days 1 to 3 at 20 g·day^−1^) of supplementation prior to saturation of muscle Cr stores [19], but may be more relevant at lower doses (e.g., 3–5 g·day^−1^, which takes up to 28 days to saturate). Indeed, the upper threshold of saturation across individuals appears remarkably consistent [1], meaning the dosing protocol will be important.

Exercise has also been shown to enhance Cr accrual in muscles. In the seminal study from Harris et al. (1992), various doses of Cr were given to healthy participants aged 20 to 62 years, with varying levels of fitness. An additional aim of this study was to determine the effect of exercise upon Cr uptake into muscles using a unilateral leg exercise model. Throughout supplementation, participants performed 1 h of cycling exercises in one leg, while the other leg rested. Results showed that exercise potentiated the resultant increase in intramuscular Cr, with greater increases in the exercised versus non-exercised leg. These were the first data to suggest that exercise may influence the Cr loading of muscles with supplementation. These data were subsequently supported by a further study that showed a 68% greater increase in total creatine content following supplementation when a single-leg exercise (cycling at 60–70% of maximal heart rate until exhaustion) was performed [20] (Robinson et al., 1999). Thus, exercise appears to enhance the accrual of intracellular TCr with Cr supplementation, although, again, the importance thereof will likely be linked to the daily dose employed. Since high doses (20 g·day^−1^) lead to saturation in as little as 5 days, it seems unlikely that quicker loading will have much impact. However, should supplementation occur more gradually, as with doses of 3–5 g·day^−1^ over 28 days, then faster loading might incur earlier and greater benefits. It must be acknowledged that it is unclear if these studies showed an increased uptake of Cr into muscle, or an increased Cr retention. Logic would suggest that it is likely reflective of an increased muscle uptake although this should be mechanistically confirmed.

The influence of exercise on Cr loading is apparent; however, more recent investigations have suggested that the timing of Cr supplementation in relation to the exercise bout may be important, too [11,12,21,22]. To better understand why timing of supplementation in relation to exercise might be important, it is important to appreciate how exercise might enhance Cr uptake, which will be discussed in the following section.

## 3. How Creatine Timing around Exercise May Influence Subsequent Loading

### 3.1. Creatine Concentration in the Bloodstream and Training Duration

**Figure 1:**
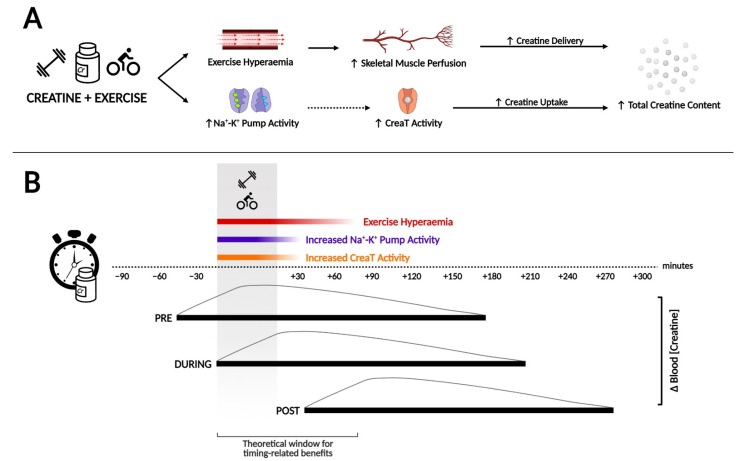
The hypothetical mechanisms behind an exercise-mediated increase in total creatine content with creatine supplementation. (**Panel A**): exercise hyperaemia increases tissue perfusion, enhancing creatine delivery. Additionally, increased Na+/K+ pump activity during exercise supports the [Na^+^] gradient favouring creatine uptake by CreaT. Together, these effects may acutely potentiate the uptake and increase in total muscle creatine content. (**Panel B**): theoretical overlap of events according to the timing of creatine supplementation, in relation to exercise and its potential benefits regarding the delivery and uptake of creatine to the muscle. Created with BioRender.com.

The mechanisms via which exercise may increase Cr uptake into muscles are not entirely understood and are hypothetical, as no study has experimentally demonstrated the mechanism behind this phenomenon. Nonetheless, one proposed mechanism via which timing of Cr ingestion in relation to exercise may modify the efficacy of supplementation is through exercise hyperaemia, namely increased blood perfusion to the working muscle (Figure 1A). Blood flow increases within one second of the onset of muscular contraction, and exercise can increase skeletal muscle blood flow by 100-fold compared to values seen at rest [23]; it is important to maintain adequate oxygen and nutrient delivery, in order to support the energetic demands of the skeletal muscles during exercise. The extent to which blood flow increases during and after exercise is influenced by factors such as the duration, type, intensity, and volume of exercise. This is important to note because muscle blood flow is closely matched to the metabolic demands of contractions induced by the exercise [24]. Theoretically, greater blood flow to the muscle could lead to greater delivery of Cr and, thus, enhance its uptake and retention, although this would primarily be restricted to the exercised muscles. An increase in blood flow kinetics as well as Cr transport to exercised muscles can result in greater delivery, retention, and metabolization of the nutrients to the exercised muscles [25]. Thus, if supplementation is provided around exercise, then circulating Cr could coincide with increased blood flow to the muscle (Figure 1B).

An important factor to consider, in regard to the timing of Cr supplementation in relation to exercise, is the time it takes for the Cr concentration to become elevated in the bloodstream. This is relevant to determine if ingestion of Cr pre- or post-exercise would provide distinct elevations of intramuscular Cr. In humans, Cr is actively absorbed from the gastrointestinal tract before entering the bloodstream to be delivered to various tissues throughout the body [26]. Creatine monohydrate absorption is close to 100% [27], and when 2 g of Cr is consumed in an aqueous solution, it reaches peak plasma concentration in approximately 1 h. This is similar to other protocols in which maximum plasma concentration of Cr occurred in <2 h when the dose administered was <10 g [18,28,29]. Although higher doses >10 g can take up to 2.5 to reach peak concentration in the blood [30], the most employed single-dose of 5 g should peak around 1–2 h following ingestion, remaining elevated for a further 4 h [5,30]. This information could be crucial when optimizing supplement timing; if Cr-uptake is maximised when there is increased muscle blood flow, then individuals should look to coincide peak circulating Cr levels with hyperaemia.

As an example, the duration of a typical resistance training session varies between 40 and 90 min, and elite bodybuilders reported an average of 60–70 min per training session [31]. Thus, should an individual supplement immediately pre-exercise or even during exercise, Cr would begin to accumulate throughout the training session, and it is possible that peak Cr concentration in the blood would still occur during exercise. Due to the exercise undertaken, this would lead to increased blood flow to the working muscles, which may lead to increased delivery and subsequent uptake of Cr, explaining, at least partially, the greater increases in Cr loading shown previously [5,30]. This also suggests that pre-exercise Cr supplementation may be more effective at increasing muscle Cr content than post-exercise supplementation. Increased blood flow to the muscles can decrease within the following 30 min after exercise [32], although the magnitude of the post-exercise hyperaemia is proportional to the strength of the contraction and its duration [33]. Depending on the modality, intensity, and duration of the exercise (i.e., muscle contraction), vasodilation may continue for up to 120 min post-exercise. Taken together, post-exercise supplementation may not benefit from exercise-induced muscle blood flow to the same extent as pre-exercise supplementation due to a shorter overlap between circulating Cr and exercise-induced hyperaemia (Figure 1, Panel B).

Thus, if the primary mechanism by which exercise induces an increased Cr loading of muscle is via an exercise-stimulated increase in blood flow to the working muscles, then pre-exercise Cr supplementation would be expected to be the most effective supplementation strategy compared to supplementation intra- or post-workout, or at any other moment of the day.

### 3.2. Na^+^-K^+^ Pump Activity and Exercise

Creatine transport into muscle cells is performed by a specific Cr transporter, CreaT [34]. This transport occurs against a concentration gradient and is dependent on the presence of extracellular Na^+^ [35], meaning Cr uptake is achieved via a Na^+^-Cr cotransport system, which makes use of the sarcolemmal Na^+^-K^+^ pumps [36]. Thus, one other mechanism that might optimise Cr supplementation is an upregulation of the kinetics involved in the Cr transport from the bloodstream to the skeletal muscle, via an increase in Na^+^-K^+^ pump activity during and following exercise [37]. Indeed, exercise training involving a 2-h exercise cycle per day, for 6 consecutive days at 65% of maximal aerobic power, induced upregulation in sarcolemmal Na^+^-K^+^-ATPase concentration in humans, after only one week of training, in the exercised muscle [38].

Studies have shown that the Na^+^-K^+^ pump regulates transsarcolemmal [Na^+^] and [K^+^] gradients in skeletal muscles and is critical for the maintenance of membrane excitability and contractility [20,39]. Odoom et al. [36] showed that the pharmacological activation and inhibition of Na^+^-K^+^ pump activity in mouse myoblast cells were paralleled by up- and downregulation of cellular Cr accumulation, demonstrating the relationship between Na^+^-K^+^ pump activity and Cr uptake. This mechanistic evidence suggests that increased Na^+^-K^+^ pump activity, as occurs with exercise, might lead to increased Cr uptake (Figure 1A), although it is currently speculative as to whether it occurs in humans.

Since the upregulation of muscle Na^+^-K^+^ pump function in the exercised limb facilitates muscle Cr transport, if this mechanism holds true for humans, the timing of Cr supplementation around exercise could alter the uptake into the muscle. Specifically, pre-exercise supplementation might ensure that high circulating levels of Cr coincide with peak activation of the Na^+^-K^+^ pump during exercise-induced muscle contraction, leading to greater intramuscular Cr accumulation (Figure 1B). However, there is a residual effect of exercise that could last from several minutes up to 48 h post-exercise, depending on the action of interest (e.g., insulin sensitivity; [40]), meaning that post-exercise Cr supplementation might also benefit from a contraction-induced potentiation of Na^+^-K^+^ pump activity, although this is highly speculative. Furthermore, most individuals taking Cr supplements undertake regular exercise training, which chronically upregulates Na^+^-K^+^ pump activity [38]. Thus, it is possible that timing in relation to each exercise session may not be important, but that exercise training in general leads to greater Cr accumulation in muscles due to chronic adaptations in Na^+^-K^+^ pump activity. This is somewhat in contrast to evidence suggesting that ingestion of Cr close to an exercise session leads to greater increases in TCr than supplementation that is not close to the exercise session (>5 h) [41].

Skeletal muscles appear to be highly amenable to Cr supplementation, while chronic exercise appears to further increase the response to supplementation. Nonetheless, what is unclear is whether timing around exercise also generates differential responses in the muscle Cr loading response, and subsequent performance gains. The hypothetical mechanisms discussed provide some support to suggest that exact timing, in relation to exercise, may exert differential effects. The following section will detail studies that have investigated the effects of pre-, during-, or post-workout Cr supplementation on several outcomes.

## 4. Creatine Supplementation Pre-, During- or Post-Workout: The Evidence

Cribb and Hayes [41] investigated the effects of Cr supplement timing during 10-weeks of resistance exercise training on intramuscular TCr content, muscle-fibre hypertrophy, strength, and body composition. Recreational male bodybuilders were allocated into two groups: one that consumed their supplements immediately pre- and post-workout on training days, and the other that consumed their supplements in the morning before breakfast and late evening before sleep. The supplements contained 40 g glucose, 40 g protein, <0.5 g of fat, and 7 g of Cr monohydrate per 100 g; participants consumed 1 g·kg^−1^·day^−1^ twice on training days only. The 10-week training program was performed 4 times a week and was designed specifically to increase strength and muscle size, with a progressive overload consisting of three compound exercises with free weights based on repetition maximum (RM) of the participants. The group who ingested the supplements around their workouts had greater increases in intramuscular TCr and greater gains in maximum dynamic strength, lean mass, and cross-sectional area type II fibres compared to the group who consumed Cr at alternate times of the day. These findings suggest that supplement timing can play an important role in strength and muscle gains, although the strength and muscle gains cannot be limited to Cr supplementation only, since the supplement contained various other ingredients, including a substantial amount of protein. It is known that the timing of protein ingestion around exercise may influence hypertrophy and strength gains [42]. Furthermore, carbohydrates were included in the supplement, which was shown to enhance Cr uptake into the muscle [18]; thus, the isolated effect of exercise on muscle TCr loading was not determined. Finally, supplementation was provided, both pre- and post-exercise, as well as “not close” to the exercise session, meaning no inferences can be made regarding whether supplementation pre-, during-, or post-exercise influences these responses.

Timing of Cr supplementation in relation to exercise has been suggested to influence the accrual of muscle Cr [43], which may impact subsequent performance gains. We have discussed the physiological mechanisms through which Cr timing around exercise might modify its loading effects, but it is important to determine the true impact of timing experimentally. A few studies have investigated the influence of supplement timing with Cr in relation to exercise on a number of different outcomes.

The first study that specifically investigated whether Cr supplementation around exercise modified its effects was performed by Antonio and Ciccone (2013). They investigated the effects of Cr supplementation, either immediately pre- or post-exercise, throughout resistance exercise training on body composition and muscle strength (Table 1). Nineteen healthy recreational bodybuilders were randomly assigned to one of two groups, ingesting either 5 g of Cr immediately pre-workout or 5 g of Cr immediately post-workout. Supplements were ingested according to the volunteer’s convenience on non-training days. Training consisted of resistance training 5 days a week for four weeks. Results showed greater muscle hypertrophy and strength gains when Cr was ingested post- versus pre-exercise. Specifically, post-exercise ingestion led to a 3% gain in fat-free mass and 7.5% gain in 1-RM bench press, compared to a 1.3% increase in fat-free mass and 6.8% 1-RM bench press improvement with pre-exercise ingestion. The authors concluded that consuming Cr immediately post-workout is superior to pre-workout on body composition and strength. These results somewhat contrast what might be expected, since increased plasma Cr levels will not coincide with increased blood flow that occurs during the exercise. However, it is important to note that no significant interactions were shown, and that magnitude-based inferences were used to determine possible and likely beneficial effects of timing on outcomes. Unfortunately, this analysis method has come under substantial criticism [44,45], while the absolute difference in fat-free mass and bench press gains were small, with overlapping confidence intervals. Thus, the true importance of these differences is somewhat unclear.

This first study to directly investigate the influence of timing of Cr supplementation around exercise has some important strengths, such as the dose administered, which is commonly employed by bodybuilders [46], and provides favourable outcomes to increase lean muscle mass [47]. Training frequency and volume per week were in the range frequently used by this population [48], while protein intake was high, namely 1.9 g·kg^−1^·day^−1^, which is expected to contribute to muscle strength and hypertrophy when combined with resistance training [49]. Despite these strengths, this study also has limitations, which must be considered when extrapolating the findings. First, the researchers did not analyse intramuscular Cr content, limiting the interpretation of the potential mechanisms related to the benefits observed with the timing of Cr ingestion. There was no direct and reliable measurement of skeletal muscle hypertrophy [50], which could have strengthened the findings. There was also no placebo-control group, which makes it impossible to consider the isolated influence of the resistance training session on body composition and strength, and the variations thereof. The differences shown here may well be within normal variations expected with a resistance training program. Volunteers also knew their timing of Cr supplementation, which could have created certain expectations in the participants [51]. Although protein intake was high, it is unclear when protein was ingested around exercise, which may have influenced hypertrophy and strength gains [42], while the small sample size was not conducive to clear conclusions. Finally, the authors did not report whether the athletes were familiarised with the 1-RM test used as the primary outcome measure. Since there may be a learning effect for such outcomes, and the athletes may have had different levels of familiarity with the exercise, this may have influenced the results to some extent [52]. Although the results by Antonio and Ciccone [11] suggest that Cr supplementation post-workout may provide greater gains in muscle strength and fat-free mass compared to pre-exercise Cr, the aforementioned limitations preclude solid recommendations without further supporting evidence.

Candow et al. [22], following the pioneering work by Antonio and Ciccone [11], compared the effects of Cr supplementation ingested immediately before vs. immediately after supervised resistance training in healthy older adults (Table 1). The 22 participants, who were not previously engaged in resistance training, were randomised in a double-blind design to one of two supplementation groups: one received Cr before (0.1 g·kg^−1^ Cr + 0.1 g·kg^−1^ placebo after exercise) and the other after (0.1 g·kg^−1^ placebo before + 0.1 g·kg^−1^ Cr after exercise) exercise. Resistance training was performed 3 days/week, on non-consecutive days, for 12 weeks. Results showed there was no difference between groups for gains in maximum strength, increases in muscle thickness, and changes in body composition, suggesting that supplement timing of Cr does not affect these measures.

This study has some important strengths, including a 12-week intervention, which is more than enough time to verify the effects of Cr supplementation [8]. Muscle thickness was measured, an important and relevant measure to observe resistance training-induced hypertrophy. However, similar to the study by Antonio and Ciccone (2013), the protocol did not include a placebo-only group to determine the true effects of the training alone. The study also lacked measures on muscle TCr changes with supplementation and training, an important consideration, since the supplementation protocol differed somewhat to those commonly employed in the literature. Cr ingested at a dose of 20 g for 5–7 days is sufficient to saturate muscle Cr stores [5], while ~3–5 g/day of Cr for 4 weeks similarly saturates skeletal muscle Cr levels [8,53]. The Cr supplementation protocol used by Candow et al. (2014) was not assessed in this sense. Although the ingested dose on training days was similar to the latter dosing strategy (~7 g of Cr for a 70 kg individual), supplementation was only performed three times per week. The extent to which this strategy increases TCr in the initial days/weeks, or when it would saturate muscles, is unknown. Nonetheless, it is likely that this dosing strategy would lead to a slow, transient increase in muscle Cr stores, meaning the timing of Cr supplementation in relation to exercise—there might be differences during such a supplementation protocol if it truly has an impact. It is also currently unclear if these same results apply to young healthy adults since elderly adults appear to respond differently to Cr supplementation [9]. Despite these limitations, the results of this study suggest that when intermittent low doses of Cr are consumed during chronic resistance training for 12 weeks, then the timing of supplementation pre- or post-exercise does not exert differential effects on strength, hypertrophy, and body composition in healthy older adults.

The largest study to date on the topic of Cr timing involved a 32-week resistance exercise training program [12] (Table 1). Thirty-nine healthy older adults completed the double-blind placebo-controlled design, and were randomised into three groups: “Cr-Before” (0.1 g·kg^−1^ Cr immediately before +0.1 g·kg^−1^ placebo immediately after RT); “Cr-After” (placebo immediately before + Cr immediately after RT); or placebo (corn starch maltodextrin immediately before and immediately after RT). Creatine was ingested only on training days and the resistance training intervention consisted of a supervised whole-body program performed 3-days per week in which the participants completed 3 sets of 10 repetitions at an intensity corresponding to their 10-RM for each exercise. Following the 32-week intervention, both Cr groups exhibited similar strength gains, with changes greater than the placebo control group.

It is interesting to note that the group that ingested Cr immediately after the training sessions showed greater increases in lean tissue mass compared to the control group, although not compared to the group that consumed Cr pre-training. There were also no differences in lean tissue mass changes between individuals in the control group and those supplementing Cr pre-training. The authors attributed the greater improvements obtained from post-workout supplementation to a better Cr absorption kinetics [54] and an increase in skeletal muscle blood flow during resistance training, which would result in greater Cr transport and accumulation in the exercised muscles, although this appears contradictory to expectation. Pre-exercise supplementation would mean that increased plasma Cr would coincide with an increased exercise-induced blood flow, potentially enhancing Cr uptake into muscle. Therefore, the reasons for these results are unclear. However, this study is similarly limited by a lack of muscle Cr determination, which might have helped to explain, at least in part, some of the differences shown between groups. A lack of direct measurement of the changes in muscle size also limits the evaluation of the strength training-induced local hypertrophy. Again, any extrapolation of these results must be limited to the elderly population due to distinct changes in muscle Cr content with supplementation compared to younger adults [9]. Finally, the lack of a statistical difference between the pre- and post-training Cr groups does not strongly support a differential effect of Cr timing.

More recently, a study examined the effects of Cr supplementation ingested throughout exercise training. Specifically, Mills et al. [55] analysed the effects of intra-set Cr supplementation during resistance training sessions on skeletal muscle mass and exercise performance in physically active young adults, which engaged in a structured resistance training program (Table 1). Twenty-two participants were randomised in a double-blind placebo-controlled design to a group supplemented with Cr (0.0055 g·kg^−1^ following each training set, totalling 18 sets per session) or a group supplemented with a placebo (maltodextrin, 0.0055 g·kg^−1^ following each training set, totalling 18 sets per session) during six weeks of RT performed five days per week. Muscle thickness, power, 1-RM, and muscular endurance were determined pre- and post-intervention. Leg press, chest press, total body strength, and leg press endurance improved in the Cr group, with no significant changes in the placebo group. Although these results interestingly demonstrate that chronic Cr throughout resistance exercise workouts can improve strength gains, the lack of a supplementation group that ingested Cr either pre- or post-workout, or “not close” to the workout session, hinders any solid conclusions other than the fact that Cr supplementation improves strength gains with training, something that was already well-established. It would be interesting to determine whether intra-set Cr supplementation is superior to supplementation at any other moment of the day, whether it be outside of training hours, immediately pre-exercise, or immediately post-exercise.

**Figure 2:**
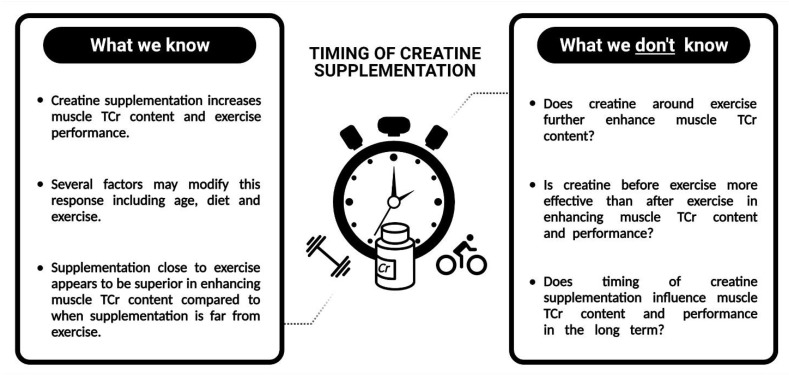
Overview of what is known about the timing of creatine (Cr) supplementation and what is yet to be determined. TCr = total creatine. Created with BioRender.com.

Currently, evidence is unclear as to whether timing of Cr supplementation around exercise modifies its efficacy (Figure 2). There is some weak evidence to support post-exercise Cr supplementation compared to pre-exercise supplementation, though the physiological mechanism underpinning these superior gains were not determined. This is of great importance since the importance of timing is only likely to have an effect during the initial phase of muscle Cr loading, and will probably be irrelevant once muscle TCr is saturated. Certainly, the limitations in the protocols highlighted herein should be considered when we try to apply the outcomes of these studies to real life, and determine whether the timing of Cr supplementation in relation to exercise is an important factor to optimize subsequent gains.

## 5. Future Directions for Research

There is evidence suggesting that Cr loading can be enhanced by exercise, with very limited data showing that Cr consumption close to exercise sessions can be more effective than ingestion in other moments of the day, at least in respect to muscle Cr loading. However, whether the timing of supplementation pre-, during-, or post-exercise has an impact is less clear. Recently, evidence suggested that post-exercise supplementation can increase muscle mass, but not strength, to a superior magnitude, when compared to pre-exercise Cr supplementation. However, considering the theoretical variables and mechanisms that influence Cr uptake into the muscles (discussed in the present review), and a series of methodological limitations presented by the available investigations, the interpretation of these results is limited. Thus, here we provide guidelines for future studies investigating this topic to ensure clarity in results and interpretation to advance our knowledge in the area.

Firstly, the lack of muscle Cr measurements in previous studies preclude solid conclusions on the efficacy of supplement timing. Without this information, it is only possible to speculate as to the effect of Cr timing in relation to exercise on changes in muscle Cr content (Figure 2). This is particularly relevant regarding the low doses of Cr typically used in these studies. It is known that a high daily dose of Cr (e.g., 20 g·day^−1^) will saturate muscle Cr content in 5–7 days [5], meaning that timing of supplementation with such a high dose will likely be unimportant. However, since lower daily doses (e.g., 5 g·day^−1^) will only saturate muscle Cr in up to 28 days [8,53], it is more than possible that enhanced loading, perhaps due to timing, might lead to greater benefits with these lower doses. Analyses during the early phases of supplementation, for example, the first 1–3 weeks with low doses (e.g., 5–7 g·day^−1^) may be most important, since measurements (once muscle Cr is already saturated) are unlikely to show any differences. Further work should ensure this measurement is included to confirm increases in muscle Cr and the extent to which it differs between timing protocols. Any subsequent changes in muscle strength, hypertrophy, or exercise performance can then be associated with these changes.

Another limitation in the current literature is related to clinical differences in the populations studied, with one study employing recreational male bodybuilders and two studies recruiting elderly non-trained populations. This is a potentially important consideration, since TCr increases in response to a standardised Cr supplementation protocol may be affected by factors, such as age, physical exercise, and diet [9]. Furthermore, small sample sizes currently hinder the strength of evidence since several studies that suggest greater improvements with one timing versus another are not supported by statistical strength [11,12]. The clinical relevance of these small differences due to timing for different populations (e.g., young vs. elderly; competitive vs. non-competitive) may be worthwhile or irrelevant. Larger population samples are required to tease out any benefits of one specific timing over another, should differences truly exist.

Further, well-designed confirmatory research is necessary to determine the magnitude of effect that Cr timing around resistance training might incur on muscle strength and hypertrophy changes with Cr supplementation. To that end, in addition to the points already raised, it would be wise to employ exercise protocols and strength measures that have previously shown robust and clear effects following Cr supplementation (e.g., 1-RM test and/or resistance training program) to quantify the size of the contribution of timing to outcomes. The use of muscular endurance tests may also be of interest since many bodybuilders frequently train until volitional fatigue, and this may be a sensitive measure to Cr supplementation [56]. Several studies appeared not to familiarise their participants to the strength tests [11,55], which is a recommendable practice for diminishing the influence of the learning effect on the strength test assessments. Including familiarisation to the exercise protocols is vital to ensure more accurate measurements. If the goal is to verify skeletal muscle hypertrophy in response to exercise and Cr supplementation, as routinely occurs with resistance exercise studies, more direct and reliable measurements of skeletal muscle hypertrophy are important assessments of exercise and resistance training-induced local hypertrophy. For example, the measurement of muscle volume or muscle cross-sectional area via ultrasound imaging, magnetic resonance imaging, or computed tomography scans would be relevant protocols to check strength training-induced local hypertrophy [57] and whether it differs according to Cr timing.

Current studies on Cr supplement timing have focussed only on timing around resistance exercise. Creatine supplementation timing should be analysed in relation to other types of exercise (e.g., repeated sprints, endurance), not only on resistance training, due to the diversity of athletes who consume this supplement regularly to improve sports performance [1]. This would allow one to determine whether the type of exercise matters to induce the contraction-stimulated uptake of Cr into the muscles, and if this is modified by timing around the activity. Again, employing exercise tests to measure performances that were previously shown susceptible to improvements with Cr supplementation might be of particular interest in this scenario. Mechanistic studies should also strive to determine whether any changes in muscle Cr content, due to timing, is due to increased uptake or increased retention via infusion and microdialysis techniques. In addition to the effects of pre- versus post-exercise timing, another possibility of ingestion of Cr is during exercise [55], so a comparison of Cr ingested during workouts and Cr consumed pre- and post-workout, and/or in other moments of the day, may be of interest. This will provide important information as to the necessity of this small-dose multi-moment ingestion strategy.

## 6. Conclusions

Although exercise appears to enhance Cr accrual in muscles with Cr supplementation, evidence supporting the importance of timing of Cr supplementation around exercise (i.e., pre- versus post- versus during-exercise) is currently limited to only a few studies. Existing data are somewhat contradictory, likely due to differing supplementation protocols, sample populations, and training protocols. As it stands, adapting Cr timing specifically, according to when training is performed, is not currently supported by solid evidence and should not be considered a real concern for now. More well-controlled studies determining whether the timing of Cr supplementation around training truly influences the increases in intramuscular Cr content and its ergogenic effects are required to substantiate any such claims.

## Figures and Tables

**Table 1:** Study protocols that investigated the timing effects of creatine supplementation before, during, and after exercise.

Reference	Population	Intervention	Outcomes
Antonio and Ciccone, [11]	19 recreational male bodybuilders.	4 weeks of 5 g·day^−1^ Cr: Group 1: Cr pre-exercise Group 2: Cr post-exercise. RT consisted of 5 d·wk^−1^ sessions.	↔ ΔBM, ΔFFM, ΔFM and Δ1-RM BP. *Possibly* (FFM, FM) and *likely* (1-RM BP) beneficial for Cr post vs. Cr pre.
Candow et al. [22]	9 men and 13 women, non-RT healthy older adults.	12 weeks of 0.1 g·kg^−1^ Cr and 0.1 g·kg^−1^ PL: Group 1: Cr before + PL after Group 2: PL before + Cr after. Cr ingested only on training days: 3 d·wk^−1^ RT session.	↔ ΔFFM ↔ ΔLimb muscle thickness ↔ Δ1-RM BP ↔ Δ1-RM LP
Candow et al. [12]	22 women and 17 men, non-RT healthy older adults.	32 weeks of 0.1 g·kg^−1^ Cr and/or 0.1 g·kg^−1^ PL: Group 1: Cr pre + PL post Group 2: Cr post + PL pre Group 3: Placebo control. Cr ingested only on training days: 3 d·wk^−1^ RT session.	ΔLBM: ↑ Cr post PL; ↔ Cr pre vs. Cr post and PL. ↔ Cr groups for 1-RM BP and LP ↑ Strength for both Cr groups compared to PL.
Mills et al. [55]	22 Physically active men and women.	6 weeks of Cr or PL post each set (intra-workout). Group 1: 0.0055 g·kg^−1^ Cr post each set Group 2: 0.0055 g·kg^−1^ Pl post each set. Cr was ingested only on training days: 5 d·wk^−1^ RT session.	↑ 1-RM BP and LP for Cr vs. PL Cr pre to post-intervention: ↑ 1-RM ↑ Muscle endurance

Abbreviations: Cr = creatine; RT = resistance training; FFM = fat free mass; FM = Fat mass; BM = body mass; RM = repetition maximum; BP = bench press; LP = leg press; LBM = lean body mass; TCr = total creatine content; ↔ = No difference; ↑ = increased; Δ = pre- to post-change.
